# Automated Electrophysiological and Pharmacological Evaluation of Human Pluripotent Stem Cell-Derived Cardiomyocytes

**DOI:** 10.1089/scd.2015.0253

**Published:** 2016-02-23

**Authors:** Divya Rajamohan, Spandan Kalra, Minh Duc Hoang, Vinoj George, Andrew Staniforth, Hugh Russell, Xuebin Yang, Chris Denning

**Affiliations:** ^1^ Division of Cancer and Stem Cells, School of Medicine, Wolfson Centre for Stem Cells, Tissue Engineering & Modelling, University of Nottingham, Nottingham, United Kingdom.; ^2^ Nottingham University Hospitals NHS Trust, Department of Cardiology, Nottingham, United Kingdom.; ^3^ Biomaterials and Tissue Engineering Group, Department of Oral Biology, University of Leeds, St. James's University Hospital, Leeds, United Kingdom.

## Abstract

Automated planar patch clamp systems are widely used in drug evaluation studies because of their ability to provide accurate, reliable, and reproducible data in a high-throughput manner. Typically, CHO and HEK tumorigenic cell lines overexpressing single ion channels are used since they can be harvested as high-density, homogenous, single-cell suspensions. While human pluripotent stem cell-derived cardiomyocytes (hPSC-CMs) are physiologically more relevant, these cells are fragile, have complex culture requirements, are inherently heterogeneous, and are expensive to produce, which has restricted their use on automated patch clamp (APC) devices. Here, we used high efficiency differentiation protocols to produce cardiomyocytes from six different hPSC lines for analysis on the Patchliner (Nanion Technologies GmbH) APC platform. We developed a two-step cell preparation protocol that yielded cell catch rates and whole-cell breakthroughs of ∼80%, with ∼40% of these cells allowing electrical activity to be recorded. The protocol permitted formation of long-lasting (>15 min), high quality seals (>2 GΩ) in both voltage- and current-clamp modes. This enabled density of sodium, calcium, and potassium currents to be evaluated, along with dose–response curves to their respective channel inhibitors, tetrodotoxin, nifedipine, and E-4031. Thus, we show the feasibility of using the Patchliner platform for automated evaluation of the electrophysiology and pharmacology of hPSC-CMs, which will enable considerable increase in throughput for reliable and efficient drug evaluation.

## Introduction

Pharmaceutical drug development is costly and time-consuming, with an average drug development duration of 10–15 years [1] and costs upward of a billion dollars [2]. Furthermore, between 1980 and 2009, approximately one in seven licensed drugs that had demonstrated sufficient efficacies in Phase III trials had to be withdrawn from the market for reasons including unanticipated side effects like cardiotoxicity, hepatotoxicity, and gastrointestinal issues [3]. Unexpected cardiotoxic side effects have been implicated in 28% of drug withdrawals in the United States [4]. It has been calculated that reducing drug attrition by 5% in Phase I clinical development can reduce drug development costs by 5.5%–7.1% [5], equating to savings of about $100 million for drug developers [6]. This has necessitated the development of various in vitro, ex vivo, and/or preclinical models to predict toxicity in humans at earlier stages of the drug development pipeline.

Phase I drug trials are commonly carried out in aneuploid tumor cell lines (eg, CHO or HEK cells) that have been genetically engineered to overexpress an ion channel of choice. However, they cannot replicate the complexity of the working cardiomyocyte, and consequently, multi-channel blocking drugs that are considered safe and “QT-neutral,” such as verapamil (dual blocking of potassium I_Kr_ and calcium I_Ca,L_ channels) are flagged as potentially harmful in the single ion channel assays [7]. Though ex vivo systems, such as ventricular wedge preparations [8] and Purkinje fibers [9], have been extensively used in physiological and pharmacological studies, their low-throughput nature and inter-species differences limit their suitability as drug screening assays. Use of animals is also not in line with the growing expectation in many countries to address the 3Rs of animal-based research (refinement, reduction, and replacement of animals) [10].

As an example of these issues, the mouse heart beats ∼10 times faster than the human heart and does not utilize the I_Kr_ (*HERG*) potassium channel, which is important in repolarization in human cardiomyocytes [11]. The downstream effects of mutations or drugs implicated in cardiac arrhythmias would ideally be studied in a primary human cardiomyocyte. However, use of such cells as large-scale pharmaceutical screens is hindered by their limited availability, poor consistency, limited proliferation, and almost immediate de-differentiation in culture [6].

Cardiomyocytes derived from human pluripotent stem cells (hPSC-CMs), comprising human embryonic stem cells (hESCs) and human induced pluripotent stem cells (hiPSCs), are now emerging as a viable alternative for drug safety testing (for reviews on the subject refer to [12,13]). Technology improvements over the last decade now allow rapid, cost-effective production of hPSC-CMs at purities that exceed 70% [14].

Even though hPSC-CMs have a relatively immature phenotype, they have shown faithful pharmacological responses to over 60 different compounds that include modulators of ion channels, β (1-, and 2-) receptors, and muscarinic receptors [6]. A blinded study that measured the effects of 12 cardiac and non-cardiac drugs over a 6-log dose–response range, covering estimated unbound therapeutic plasma concentrations, showed a close association between data obtained from hPSC-CMs and the clinic [15]. Work from GlaxoSmithKline that cross-compared the pharmacological responses of hPSC-CMs and animal models concluded that the human cells offered a reliable and cost-effective surrogate to preclinical in vitro testing [16]. hPSC-CMs were also used to show that the anticancer drug doxorubicin, delivered via an HER2-targeted liposomal pathway, reduced the cardiotoxicty observed with anthracyclines, which assisted the decision to advance the drug to Phase I testing [17].

To date, patient-specific hiPSC-CMs have been used to evaluate altered phenotype and drug rescue of various channelopathies affecting the heart, including Long QT syndrome (LQTS) 1 [18], 2 [19–22], 3 [23], 8, [24], and catecholaminergic polymorphic ventricular tachycardia (CPVT) [25,26]. Notably, Terrenoire et al. performed multi-parameter in vitro drug testing on the hPSC-CMs of a patient with complex LQTS to arrive upon a combinatorial regime that proved effective for the patient in the clinic, thus demonstrating the use of hPSC-CMs in personalized medicine [27]. Collectively, such studies have led the CIPA initiative (Comprehensive In Vitro Proarrhythmia Assay) to propose the integration of hPSC-CMs into the ICH (International Conference on Harmonisation) S7a/b and E14 guidelines by the end of 2015. These have been the mainstay over the last decade of preclinical assessment of cardiac electrophysiology for new drugs [28].

Despite these successes, virtually all studies to date have used conventional patch clamp to evaluate the electrophysiology of hPSC-CMs. Given that this approach can only produce 10–15 data points a day [29], there is now a pressing need for automated platforms that can produce high quality data at a throughput relevant to industry and academia. Automated patch clamping (APC), by allowing multiple recordings in parallel, can increase data throughput 10- to 100-fold depending on the ion channel under investigation and the platform used [30]. By reducing the complexity of the process, these platforms also make patch clamping accessible to more users, regardless of previous experience in electrophysiology. General drawbacks of parallel patch clamping include a requirement for high quality, high density, homogenous cell suspensions, which becomes restrictive when using expensive, low-yielding cell types [29].

These challenges are reflected by the paucity of data available for hPSC-CM evaluation using APCs. The PatchXpress planar patch clamp platform has been used for the APC analysis of I_Na_, I_Ca_, and I_Kr_ currents using hiPSC-CMs. However, that study used a genetically engineered hiPSC line, which allowed drug selection of the cardiomyocytes. Despite using a genetically enriched near-pure population of hiPSC-CMs, the study reported low seal rates (∼50% across 58 wells) and poor seal qualities (∼200 MΩ) [31], highlighting the need to develop better protocols for the preparation and analysis of cells by APC.

An alternative device is the Patchliner System (Nanion Technologies), which is a medium-throughput patch clamp device, capable of executing up to 48 unattended recordings and generating ∼500 data points a day [32]. However, Patchliner recordings have typically been performed on recombinant HEK and CHO cell lines, which are easy to obtain in large numbers, and form high-quality, high-density, homogenous single-cell suspensions ideal for APC (Table 1). In contrast, hPSC-CMs provide more physiologically relevant model systems for drug efficacy and safety testing (Table 1), but these are expensive to produce in large numbers and very sensitive to enzymatic processing into single-cell suspensions [6].

**Table 1.** A Comparison of the Relevance of Recombinant Cell Lines and Human Stem Cell-Derived Cardiomyocytes for Drug Screening and Development

*Attributes*	*Recombinant cell lines*	*Stem cell-derived cardiomyocytes*
**Cell line generation**		
Ease of production	Easy to grow; undergo prolonged, high-density proliferation in culture; high capacity for stable recombinant protein expression	Require specialist know-how and reagents; limited proliferation in culture; low capacity for stable recombinant protein expression
Cost of production	∼£5/L of culture media	∼£400/L of culture media
**Cell preparation for planar patch clamp**		
Homogeneity of prep	Homogeneous	Heterogeneous
Clumpiness of prep	Single cell	A mixture of single cells, cell clusters, and debris
Quality of seals	>1 GΩ	200 MΩ–1 GΩ
**Quality/relevance of data**		
Cell shape	Round	Varied
Membrane capacitance	<10 pF for HEK293	∼25 pF [29]
Multi-ion channel phenotype	No	Yes
Contractile machinery	No	Yes
Upstroke velocity	∼400 dV/dt [47]	∼17 dV/dt [45]
RMP	−40 mV [47]	∼60 mV [45]
Maximal peak I_Na_	−264 ± 34 pA/pF [47]	−216.7 ± 18.7 pA/pF [29]
Maximal peak I_Ca,L_	600 pA/pF [47]	−17.1 ± 1.7 pA/pF [29]
Peak I_Kr_ tail current	12.8 ± 1.6 pA/pF [48]	0.95 ± 0.02 pA/pF [29]

RMP, resting membrane potential.

In this report we used high efficiency differentiation protocols that do not require genetic selection to produce hPSC-CMs of purities in excess of 80% to enable optimization of the Nanion Patchliner automated planar patch clamp system across cardiomyocytes derived from six hPSC lines that include three healthy lines (one hESC; two hiPSC) and three hiPSC lines harboring disease-causing mutations (one CPVT; two Duchenne muscular dystrophy [DMD]). We developed a two-step cell preparation protocol that proved effective in enabling catch rates of 81.25% ± 6.68%, seal rates of 79.68% ± 7.06%, and seal qualities of up to >2 GΩ. Analysis in voltage clamp mode allowed evaluation of I_Na_, I_Ca,L_, and I_K_ current densities, and the impact on these currents of their respective blockers tetrodotoxin (TTX), nifedipine, and E-4031. Thus, this work paves the way to use APC for high-throughput analysis of the electrophysiology and pharmacology of hPSC-CMs.

## Materials and Methods

### Isolation of fibroblasts from patient skin biopsies

Skin punch biopsies of 4 mm were transported from the clinic into the laboratory in Transport medium (Table 2) and washed thrice in the same, after which they were transferred into bacterial-grade Petri dishes. Using sterile forceps and scalpels, the samples were cleaned off any adipose and epidermal tissue, minced into 1 mm pieces and then digested—first with 2.5% trypsin (Invitrogen) for 20 min, and then with 1 mg/mL collagenase IV (Invitrogen) for 90 min, both at 37°C. The digested cells were spun down at 200 *g* for 5 min, plated in Chang's D medium (Table 2), and grown for 2–3 weeks until confluent, with medium changes every 3–4 days.

**Table 2.** Media Formulations for Stem Cell Derivation, Culture, and Differentiation

*Acronym*	*Component*	*Conc.*	*Supplier*
Transport media	HBSS (10×)	10%	Invitrogen
	H_2_O	88%	Sigma-Aldrich
	Pen/strep	1%	Invitrogen
	Fungizone	1%	Invitrogen
Chang's D media	Chang's D	98%	Invitrogen
	L-glutamine	2 mM	Invitrogen
	HEPES	100 mM	Invitrogen
	Pen/strep	1%	Invitrogen
	Fungizone	1%	Invitrogen
Fibroblast media	1× DMEM	78%	Invitrogen
	FBS	20%	Perbio
	NEAA	1%	Invitrogen
	L-glutamine	1%	Invitrogen
	β-mercaptoethanol	100 μM	Sigma-Aldrich
hPSC media	DMEM F12	83%	Invitrogen
	KSR	15%	Invitrogen
	NEAA	1%	Invitrogen
	GlutaMAX	1%	Invitrogen
	β-mercaptoethanol	100 μM	Sigma-Aldrich
	bFGF	8 ng/mL	R&D
RIP media	1× RPMI	98%	Invitrogen
	ITS	1%	Gibco
	Chemically defined lipid	1%	Gibco
	1-Thioglycerol	400 μM	Sigma-Aldrich
	PVA	4 mg/mL	Sigma-Aldrich
	bFGF	6 ng/mL	Peprotech
	BMP4	20 ng/mL	R&D
RD media	1× RPMI	80%	Invitrogen
	FBS	20%	Perbio
	1-Thioglycerol	400 μM	Sigma-Aldrich
RB media	1× RPMI	98%	Invitrogen
	50× B-27 supplement	2%	Invitrogen

### Generation of patient-specific hiPSCs

The protocol followed for the generation of hiPSCs was based on a previously published method [33], and media formulations are as detailed in Table 2. In brief, on the day of transduction, fibroblasts were seeded into tissue culture-treated six-well plates in fibroblast medium, at a density of 50,000 cells/well. Once the cells had adhered to the plastic (after 5–6 h), they were infected with streptavidin-conjugated viral particles expressing *OCT4*, *NANOG*, *SOX2*, and *LIN28*, in the presence of 8 μg/mL of Polybrene (Sigma-Aldrich) at a multiplicity-of-infection of 10. Twenty-four hours post-transduction (p.td), the cells were washed twice with PBS and fed with fresh fibroblast medium. Forty-eight hours p.td., the cells were harvested using 0.05% trypsin (Invitrogen), resuspended in hPSC media supplemented with 10 ng/mL FGF (Peprotech), and transferred to tissue culture treated 90 mm Petri dishes containing mitomycin-C inactivated mouse embryonic fibroblasts (MEFs) at a density of 1.8 × 10^6^ cells/well.

For the first week, cells were maintained in hPSC medium containing 10 ng/mL FGF, after which the medium was switched to MEF-conditioned hPSC medium supplemented with 10 ng/mL FGF. In either case, medium changes were performed daily until around day 25-day 30 p.td.

### Cardiac differentiation of hiPSCs

#### Embryoid body-mediated differentiation

The protocol followed was based on a previously published protocol [34], and all media formulations are detailed in Table 2. In brief, on day 0 of differentiation, embryoid body (EB) formation was initiated by the seeding of hPSCs into 96-well V-bottomed plates (Thermo Scientific), at a density of 6,000 cells/well, in RIP medium. On day 2, the RIP medium was replaced with RD medium. On day 4, the EBs were transferred into 96-well U-bottomed tissue culture-treated plates (Fisher Scientific) and maintained in RB medium from thereon, with medium changes every 3–4 days. Spontaneously beating clusters were observed from day 8 onward.

#### Monolayer-mediated differentiation

Undifferentiated hPSCs were seeded onto Matrigel™-coated dishes at a density of 1 × 10^5^ cells/cm^2^, and allowed to expand for 72 h until confluent. At this stage, (day 1 of differentiation) cells were treated with medium A consisting of StemPro-34 (Life Technologies) medium, recombinant human Activin A (Life Technologies) and recombinant human BMP4 (R&D Systems). Media exchange was performed on day 3 with medium B- consisting of RPMI base medium (Life Technologies) supplemented with 1× B-27 (Life Technologies) and a small molecule inhibitor, KY02111 (R&D Systems). From day 5 onward, cells were maintained in RPMI base medium supplemented with B-27 only, and media changes were performed every 2–3 days.

### Electrophysiology

Manual patch clamp was performed using an ECP-10 HEKA amplifier. Cells were maintained in NT Buffer and at 37°C during recordings. Patch pipettes were pulled on a Sutter P-97 programmable micropipette puller and had resistances of between 2 and 5 MΩ when filled with ionic solution. Data were recorded using the Pulse software (HEKA) and analyzed using Clampfit v9.0 (Molecular Devices).

Automated electrophysiological analysis of the hPSC-CMs was performed on an NPC-16 Patchliner Octo platform (Nanion Technologies) fitted with two EPC-10 quadro amplifiers (HEKA Elektronik GmBH) using disposable borosilicate glass chips (Nanion Technologies). When filled with intracellular solution the chip resistance was between 1.8–3 MΩ. Data were acquired using the Patchmaster software (HEKA Elektronik GmBH) analyzed offline using IGOR-Pro (WaveMetrics, Inc.).

### Patch clamp solutions

Internal solutions for (1) voltage-gated Na and Ca channel recordings: 50 mM CsCl, 10 mM NaCl, 60 mM CsF, 20 mM EGTA, 10 mM HEPES, CsOH pH 7.2, osmolarity 285 mOsmol; (2) voltage-gated K channel recordings: 50 mM KCl, 10 mM NaCl, 60 mM KF, 20 mM EGTA, 10 mM HEPES, KOH pH 7.2, osmolarity 285 mOsmol; (3) action potential (AP) recordings: 145 mM KCl, 5 mM NaCl, 2 mM CaCl_2_, 2 mM MgCl_2_, 4 mM ethylene glycol tetra-acetic acid, 10 mM HEPES, KOH pH 7.3. External solutions for (1) voltage-gated K and Na channel recordings: 140 mM NaCl, 4 mM KCl, 1 mM MgCl_2_, 2 mM CaCl_2_, 5 mM D-glucose monohydrate, 10 mM HEPES, NaOH pH 7.4, osmolarity 298 mOsmol; (2) voltage-gated Ca channel recordings: 125 mM TEA-Cl, 20 mM BaCl_2_, 10 mM D-glucose monohydrate, 10 mM HEPES/TEA OH pH 7.35, osmolarity: 300–305 mOsmol; (3) AP recordings: 140 mM NaCl, 4 mM KCl, 1 mM MgCl_2_, 1.8 mM CaCl_2_, 10 mM D-glucose monohydrate, 10 mM HEPES; Seal enhancer: 80 mM NaCl, 3 mM KCl, 10 mM MgCl_2_, 35 mM CaCl_2_, 10 mM HEPES (Na^+^-salt), HCl pH 7.4, osmolarity: 298 mOsmol

### Ethics

This study conformed to the principles outlined in the Declaration of Helsinki. All subjects gave informed consent for blood testing for genetic abnormalities associated with hereditary cardiac channelopathies, and for tissue donation. Isolation and use of patient fibroblasts was approved by the Nottingham Research Ethics Committee (Approval 09/H0408/74), and sample collections are registered with the U.K. Clinical Research Network under IRAS project ID24624. Isolation and use of patient dental pulps were also performed with full patient consent and ethical approval (LREC 180808/HR/12).

### Statistics

Statistical significance was determined by Student's *t*-tests, or ANOVA with Tukey post-hoc tests for pairwise comparisons (MS Excel 2010). Data were considered significant where *P* < 0.05. Results are presented as mean values ± standard error of mean; *n* denotes the number of cells in which measurements were made.

## Results

### Generation and characterization of hPSC-CMs

Two healthy hiPSC lines [HUES7-fibroblast-derived FIB-hiPSC and dental pulp-derived BT1-hiPSC], and three diseased hiPSC lines (DMD-afflicted DMD4- and DMD16-hiPSCs and CPVT-afflicted CP1-hiPSCs) were generated in vitro by lentiviral delivery of the *OCT4*, *NANOG*, *SOX2*, and *LIN28* reprogramming factors. These putative hiPSC lines were measured against internationally accepted pluripotency criteria to establish their phenotype [35].

**FIG. 1.**
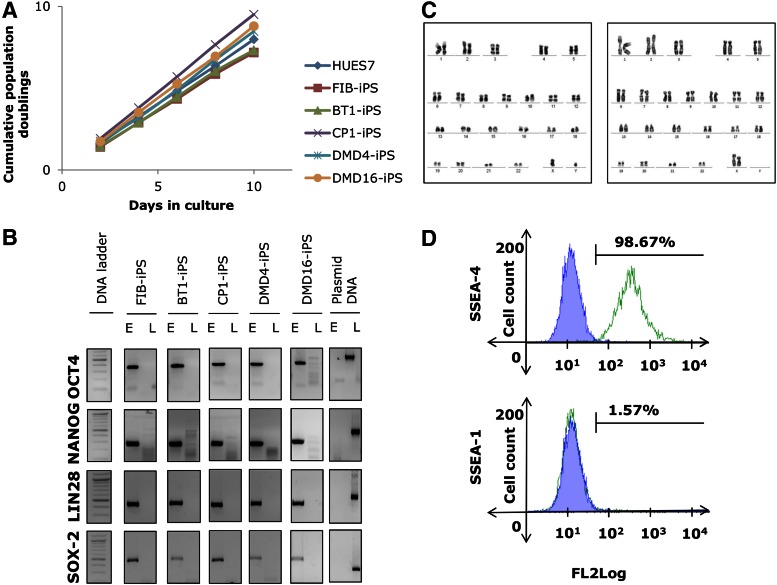
Characterization of hiPSC lines. **(A)** PDs at each passage were calculated using the formula [log10 (total cell counts/cells seeded)/log10 (2)], where the cell number seeded was 48,000 cells/cm^2^. All five iPSC lines exhibited PDs similar to that of HUES7 hESCs, ∼33.5 h. **(B)** RT-PCR analysis of *OCT4*, *NANOG*, *SOX2*, and *LIN28* expression from endogenous “E” and lentiviral “L” loci in FIB-, BT1-, CP1-, DMD4-, and DMD16-iPS demonstrating the activation of endogenous pluripotency genes and the silencing of lentiviral transgenes, at passage 15. **(C)** Representative karyograms showing normal 46XY and 46XX karyotypes, at passage 15. **(D)** Representative flow cytometry data of FIB-iPSCs demonstrating the expression of pluripotency marker SSEA-4, and the absence of differentiation marker SSEA-1. DMD, Duchenne muscular dystrophy; FIB, HUES7-fibroblast-derived; hESCs, human embryonic stem cells; hiPSC, human induced pluripotent stem cells; PD, population doublings. Color images available online at www.liebertpub.com/scd

Karyotypic analyses revealed that the cells were genetically stable with a normal complement of 46XY or 46XX chromosomes (Fig. 1C), and their population doubling times were similar to those of HUES7 hESCs (Fig. 1A). RT-PCR analysis demonstrated that the hiPSCs had reactivated the reprogramming factors *OCT4*, *NANOG*, *SOX2*, and *LIN28* at their endogenous loci, and silenced the lentiviral transgenes (Fig. 1B and Supplementary Fig. S1; Supplementary Data are available online at www.liebertpub.com/scd). Immunostaining confirmed that the hiPSCs had silenced the fibroblast-specific marker FSA; and reactivated the pluripotency markers TRA-1-81, SSEA-4, OCT4, and LIN28 (Supplementary Fig. S2). Flow cytometry further confirmed that the hiPSCs had silenced the differentiation marker SSEA-1; and reactivated the pluripotency marker SSEA-4 (Fig. 1D and Supplementary Fig. S3). These results confirmed the pluripotent nature of the five iPSC lines.

**FIG. 2.**
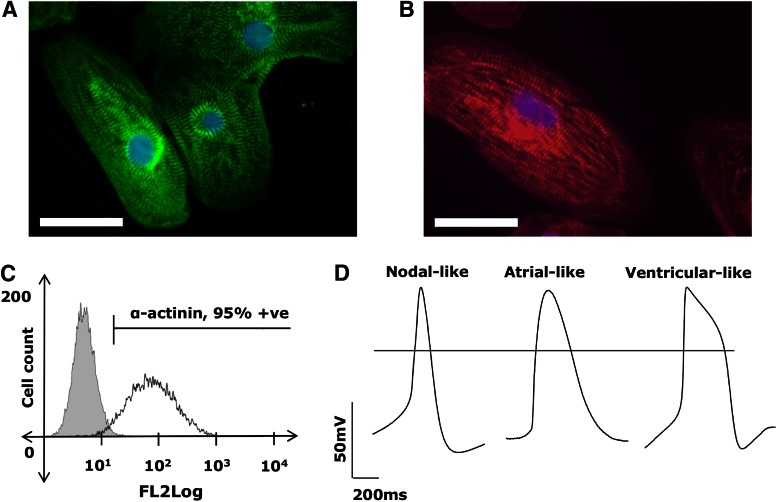
Characterization of hiPSC-derived cardiomyocytes (hiPSC-CMs). Representative immunofluorescence images of HUES7-cardiomyocytes showing positive **(A)** cardiac α-actinin, and **(B)** cardiac troponin T staining highlighting characteristic cardiac muscle striations. Scale bars represent 32 μm. **(C)** Representative flow cytometry data of HUES7-CMs demonstrating cardiac purities of 91.2% ± 1.4% (Supplementary Fig. S6). **(D)** Representative AP traces demonstrating the formation of atrial-like, ventricular-like, and nodal-like cardiomyocytes. The classification of different cardiac cell types was based on the APD_90_/APD_50_ ratio. APD_90/50_ values ≤1.4 were designated as ventricular-like, 1.4–1.7 were designated as nodal-like, and ≥1.7 were designated as atrial-like. AP, action potential. Color images available online at www.liebertpub.com/scd

To generate human cardiomyocytes in vitro, these five hiPSC lines along with a HUES7 hESC control were differentiated via the embryoid-body and monolayer-routes (Supplementary Videos S1 and S2). The presence of cardiac-specific proteins and their spatial organization within the hPSC-CMs were determined by dissociating the beating clusters/sheets and staining the dispersed cells with immunofluorescent cardiac α-actinin and cardiac troponin T (cTnT) antibodies (Fig. 2A, B and Supplementary Figs. S4 and S5). All six lines produced hPSC-CMs with similar morphologies and patterns of myofilament organization. While cTnT appeared as longitudinal, punctuate, fibrous striations, a hallmark of functional cardiomyocytes, α-actinin appeared homogenously throughout the cell as longitudinal fibrous striations. The hPSC-CMs had multi-angular morphologies appearing round, elongated, branched, or triangular, with one or more oval nuclei. Flow cytometry analysis for the cardiac α-actinin marker revealed the cardiac preparations to have purities of 91.2% ± 1.4% (Fig. 2C and Supplementary Fig. S6).

To assess the electrophysiological functionality of the hPSC-CMs, beating clusters or monolayers of cardiomyocytes were disaggregated into single cells, seeded onto MatTek dishes, and analyzed by whole-cell patch clamp. The APs recorded from these cells demonstrated, on average, AP duration (APD), APD_50_ (APD at 50% of the repolarisation curve), APD_90_ (APD at 90% of the repolarization curve), AP amplitude (APA), resting membrane potential (RMP), and upstroke velocity values of 394.9 ± 27.6 ms, 106.7 ± 12.2 ms, 154.9 ± 15.7 ms, 55.8 ± 4.1 mV, −33.2 ± 3.4 mV, and 6.3 ± 1.5 V/s respectively. Table 3 lists the specific values obtained for the AP waveforms of HUES7- (*n* = 58), FIB-iPS- (*n* = 44), and BT1-iPS (*n* = 29)-derived cardiomyocytes.

**Table 3.** Action Potential Parameters of Human Pluripotent Stem Cell-Cardiomyocytes as Determined by Manual Patch Clamp

*Cell type*	*Cell line*	*Cslow (pF)*	*RMP (mV)*	*APA (mV)*	*Upstroke velocity (V/s)*	*APD (ms)*	*APD_50_ (ms)*	*APD_90_ (ms)*
Atrial-like	HUES7	34.1 ± 11.1	−29.2 ± 14	44.9 ± 7.1	5.6 ± 10.9	429.3 ± 2.3	84.7 ± 4.5	165.2 ± 3.5
	FIB-iPS	26.5 ± 5.1	−35.23 ± 2.4	44.5 ± 4.2	5.9 ± 1.6	518 ± 129.3	105.3 ± 27.9	192.3 ± 48.8
	BT1-iPS	29.2 ± 5.9	−22.3 ± 1	35.1 ± 3.8	2.5 ± 0.5	379.1 ± 42.9	49.4 ± 7.6	95 ± 16.1
Ventricu lar-like	HUES7	19.4 ± 4.3	−36.2 ± 3	68.8 ± 5.8	1.9 ± 0.5	327.2 ± 18.1	120.3 ± 10.3	149.8 ± 10.5
	FIB-iPS	14.8 ± 3.8	−53.3 ± 5.1	62.7 ± 3.7	13.3 ± 3.7	336.3 ± 50	158.5 ± 27.3	127.3 ± 22.3
	BT1-iPS	21.8 ± 4	−25.4 ± 2.9	69.7 ± 4	12.1 ± 2.4	252.5 ± 21	59.8 ± 4.5	77.7 ± 6
Nodal-like	HUES7	29.8 ± 5.5	−23.1 ± 2.3	63.5 ± 3.7	9.9 ± 2	447.4 ± 24.6	115.5 ± 7.7	183.6 ± 12.2
	FIB-iPS	10.2 ± 1.6	−42.5 ± 3.8	50.4 ± 5.9	3.2 ± 0.9	479.3 ± 100.8	145.9 ± 30.4	220.9 ± 46.1
	BT1-iPS	14.6 ± 2	−31.9 ± 3.1	62.4 ± 8.3	1.9 ± 0.4	384.6 ± 86.4	121.1 ± 52.6	182 ± 78.5

The APD_90_/APD_50_ ratio, which describes the shape of the repolarization curve, was used to determine the specific subtype of each hPSC-CM analyzed. hPSC-CMs with low APD_90_/APD_50_ ratios (≤1.3, indicative of a pronounced plateau phase) were classified as ventricular-like, while those with high APD_90_/APD_50_ ratios (≥1.8, indicative of fast phase-I repolarization) were classified as atrial-like. Cardiomyocytes with AP characteristics intermediary to those of the atrial-like and ventricular-like cells, and APD_90_/APD_50_ ratios between 1.4 and 1.7 were classified as nodal-like [20]. The HUES7, FIB-iPS, and BT1-iPS lines all produced cardiomyocytes of atrial-like, ventricular-like, and nodal-like morphologies (Table 3), with representative traces shown in Fig. 2D.

Thus, the structural and electrophysiological properties of hPSC-CMs were consistent with other reports describing these cells, which permitted their use in development of protocols for automated electrophysiology analysis.

### Development of a cell harvesting protocol compatible with automated planar patch clamp

**FIG. 3.**
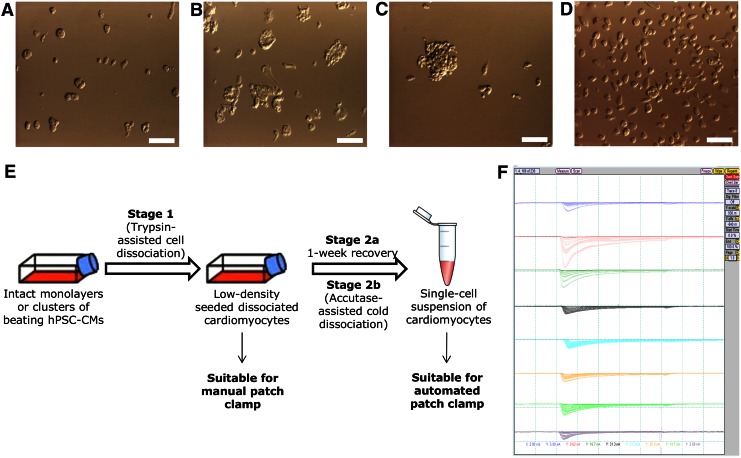
Development of a cell harvesting method compatible with automated planar patch clamp. **(A)** The dissociation of intact embryoid bodies or monolayers with trypsin produced single cells with little/no currents (Table 4); Collagenase/accutase/dispase treatment **(B)** and mechanical dissociation protocols **(C)** produced cell clumps unsuitable for efficient planar patch clamping. **(E)** The two-step cell dissociation protocol presented in the study produced **(D)** single cells with functional currents suitable for efficient analysis of hPSC-CMs on the Patchliner. **(F)** Shown are the raw current traces on a chip where 100% catch rates and whole-cell breakthroughs were achieved with HUES7 hESC-CMs using the newly developed two-step cell dissociation protocol **(E)**. hPSC-CMs, human pluripotent stem cell-derived cardiomyocytes. Color images available online at www.liebertpub.com/scd

As with any planar patch clamp platform, the Patchliner requires freshly dissociated single cells in suspension. To achieve this, intact clusters or monolayers of hPSC-CMs of 91.2% ± 1.4% purity (Fig. 2C and Supplementary Fig. S6) were dissociated into single cells using a trypsin-containing cell dissociation solution at 37°C (Fig. 3A). Trypsin was included in the dissociation solution as it was the only enzyme we found capable of breaking the tightly clustered cardiomyocytes within EBs and monolayers into single cells. Other “gentler” enzymes like accutase, collagenase, and dispase produced cell clumps that were unsuitable for planar patch clamp (Fig. 3B). However, when processed on the Patchliner, trypsin-treated cells demonstrated low catch rates of 54.16% ± 11.89%, and a low incidence of conversion to whole-cell breakthroughs (34.72% ± 8.17%). Furthermore, only 8.33% ± 4.7% of the cells that were successfully patched exhibited functional currents (Table 4). The cardiac differentiation of hPSCs is a costly, time-consuming, and labor-intensive process, and the derived cardiomyocytes are fragile, highly sensitive to the environment, and characterized by low (ie, physiological) levels of endogenous ion channel expression (Table 1). To minimize cell wastage and improve the productivity of the Patchliner, we set about developing a cell preparation protocol that allowed optimal use of our hPSC-CMs on the Patchliner.

**Table 4.** Development of a Cell Harvest Protocol Optimal for Analysis on the Patchliner

*Protocol*	*% catch rate*	*% whole-cell*	*% cells showing APs or currents*	*Cell quality*	*Comments*
Trypsin-containing solution	54 ± 12	35 ± 8	8 ± 5	Single cells (Fig. 3A)	Enzyme treatment damaging cells
Mechanical dissection	—	—		Cell clusters and debris (Fig. 3C)	No single cells and cell clumps/debris clogging wells
Cold dissociation	69 ± 11	58 ± 10	5 ± 2	Single cells	Better cell viability and membrane integrity, but no currents
Two-step protocol	81 ± 7	80 ± 7	31 ± 6	Single cells (Fig. 3D)	Gentler the enzyme treatment on the day of analysis, better the quality of the recordings

Reasoning that the inclusion of trypsin was damaging the cell membrane [36] and reducing the density of functional ion channels, we evaluated whether hPSC-CMs could be successfully prepared by mechanical dissection using a stem cell knife, a commercially available glass capillary with a 200 μm bore, and a sharpened 45° cutting edge. However, while this approach broke the cardiomyocyte clusters into clumps, it did not yield single cells (Fig. 3C). When these “minced” cell preparations were run on the Patchliner, the clumps blocked the holes of the planar chip preventing any successful recordings (Table 4). As an alternative, we tested a trypsin-including cold dissociation protocol [37] to try and better preserve the integrity of the cell membranes. While this method improved catch rates to 69.44% ± 10.85%, the single cells patched showed little to no currents, suggesting that channel degradation due to enzymatic treatment still remained an issue (Table 4).

To evolve the protocol further, we separated the dissociation protocol into two steps. At least 1 week before use on the Patchliner, hPSC-CM cultures were dispersed with trypsin-containing cell dissociation solution. This relatively harsh treatment produced a single-cell suspension of hPSC-CMs that could be reseeded at a low density onto gelatine-coated tissue culture plastic, with cells recovering their ability to beat spontaneously within 3 days of the procedure. A week after this preseeding step, hPSC-CMs were reharvested using a gentle accutase-assisted cold dissociation protocol to produce a single-cell suspension that was used immediately for Patchliner analysis (Fig. 3D). Relative to the earlier approaches used, this two-step method yielded significant (*P* < 0.05, *n* = 5 in each treatment group) improvements in catch rates (81.25% ± 6.68%), whole-cell breakthroughs (79.68% ± 7.06%), and percentage of cells showing APs or ionic currents (31.25% ± 5.79%) (Table 3). In some cases, all eight channels of the Patchliner chips gave productive recordings (Fig. 3F). Therefore, this approach to hPSC-CM preparation (Fig. 3E) was used in all subsequent electrophysiological and pharmacological analyses on the Patchliner.

### Study of hPSC-CM APs by automated current-clamp

**FIG. 4.**
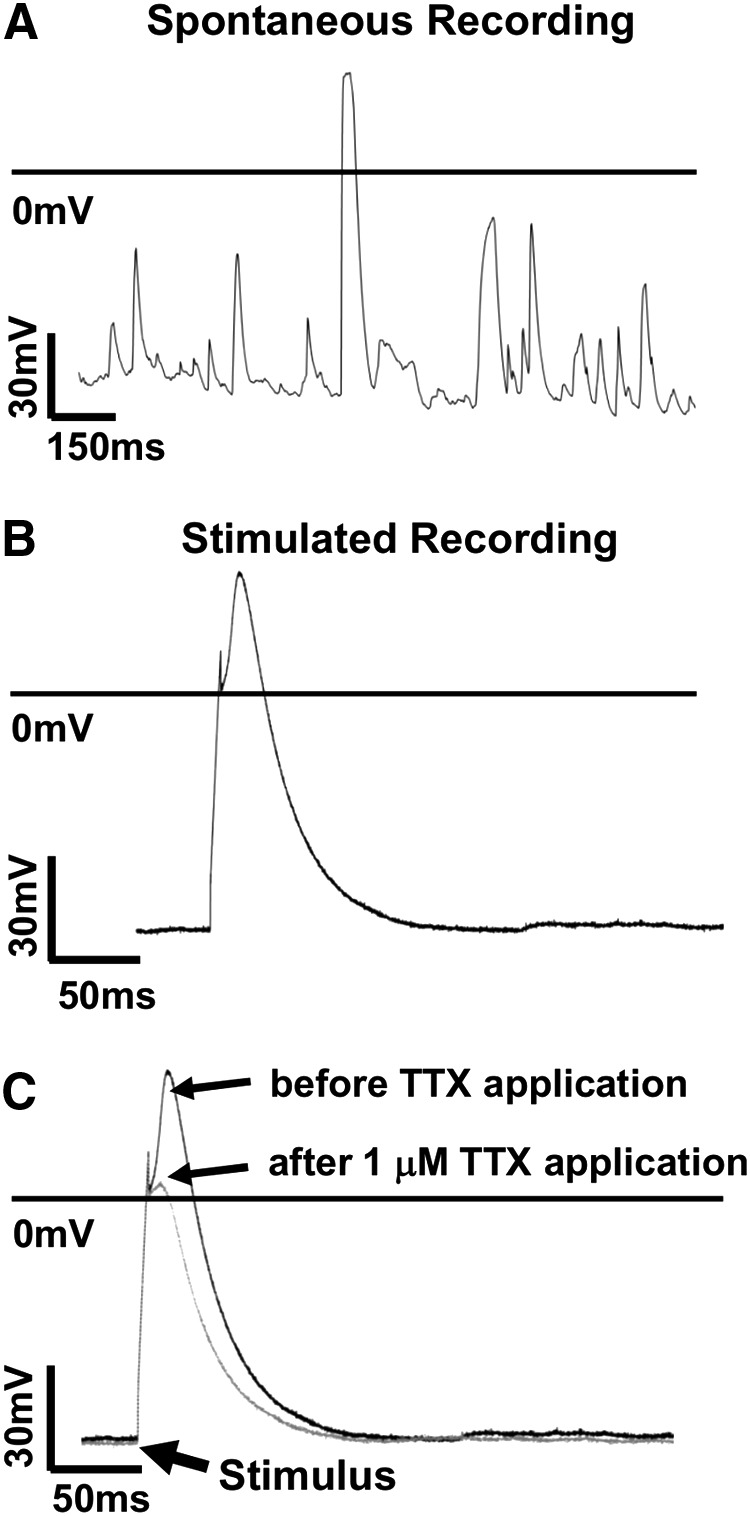
Automated electrophysiological analysis of the hPSC-CMs in current clamp mode. Representative raw data traces of **(A)** spontaneous APs from HUES7-CMs showing an erratic pattern of activity with individual spikes rarely exceeding 20 ms in duration. **(B)** In contrast, stimulation protocols produced classical AP traces. Using this approach we estimated that the cells had APD_90_, upstroke velocity and APA values of 231.4 ± 69 ms, 11.9 ± 1.5 V/s, 96.6 ± 7.5 mV respectively (*n* = 42). **(C)** HUES7-CMs when treated with 1 μM TTX (blocker of the fast voltage-gated sodium channel I_Na_), showed a 1.6 ± 0.4-fold reduction in AP amplitude (*n* = 8), which was restored on washout with external recording solution. TTX, tetrodotoxin.

We next sought to evaluate whether the Patchliner could be used in current clamp mode to record and measure spontaneous and stimulated APs from hPSC-CMs. Following the differentiation and harvest of cardiomyocytes from the hESC HUES7 line, spontaneous APs were recorded from the cells under zero current clamp conditions. However, despite stable seal properties, most cells were electrically quiescent, and any spontaneous activity recorded was erratic and unfit for analysis (Fig. 4A). Based on the data above (Fig. 2, Supplementary Fig. S2–S6 and Table 3), this was not a cell quality issue but rather the requirement for cells in suspension at the time of analysis and storage at 4°C before use.

To determine whether the data quality could be improved by pacing, preparations of HUES7-CMs were subjected to electrical stimulation. An AP can only be initiated when the cell membrane is hyperpolarized beyond a certain value called the “threshold potential,” which varies from cell to cell, but is constant for a given cell at rest [38]. APs were evoked from the HUES7-CMs by subjecting them to a train of 500 ms long depolarizing pulses (Δ20pA) from a holding potential of −70 mV, and analyzing their voltage responses. A depolarizing pulse that caused an amplitude rise >30 mV above RMP was defined as the “threshold current” and was subsequently used to generate APs for analysis (Fig. 4B). Using this approach we estimated that the cells had APD_90_, upstroke velocity and APA values of 231.4 ± 69 ms, 11.9 ± 1.5 V/s, 96.6 ± 7.5 mV respectively. Table 5 details cell line-specific values for HUES7- (*n* = 14), FIB-iPS- (*n* = 15) and BT1-iPS (*n* = 13)-derived cardiomyocytes.

**Table 5.** Action Potential Parameters of Human Pluripotent Stem Cell-Cardiomyocytes as Determined by Automated Planar Patch Clamp

*Cell line*	*Cslow (pF)*	*APA (mV)*	*Upstroke velocity (V/s)*	*APD (ms)*	*APD_50_ (ms)*	*APD_90_ (ms)*
HUES7	16.8 ± 3.6	83.3 ± 8.5	10.5 ± 2.3	369.8 ± 130.6	106.2 ± 45.3	177.8 ± 68
FIB-iPS	35.9 ± 21.4	93.8 ± 11.5	10.4 ± 1.9	511.9 ± 256.8	202.7 ± 139.1	282.1 ± 174
BT1-iPS	22.5 ± 3.8	115.1 ± 18	15.1 ± 3.6	417.4 ± 146	135.8 ± 66	238.6 ± 102.4

To determine the effects of drug compounds on the AP parameters of HUES7-CMs, the depolarizing Na^+^ current was inhibited by the selective I_Na_ channel blocker TTX. This caused a 1.6 ± 0.4-fold decrease in APA (*n* = 8), which was restored on washout with external recording solution (Fig. 4C). Thus it was shown that the Patchliner could be used in current clamp mode to investigate the electrophysiology and pharmacology of hPSC-CMs, although only with pacing.

### Study of hPSC-CM voltage-dependent currents by automated voltage-clamp

**FIG. 5.**
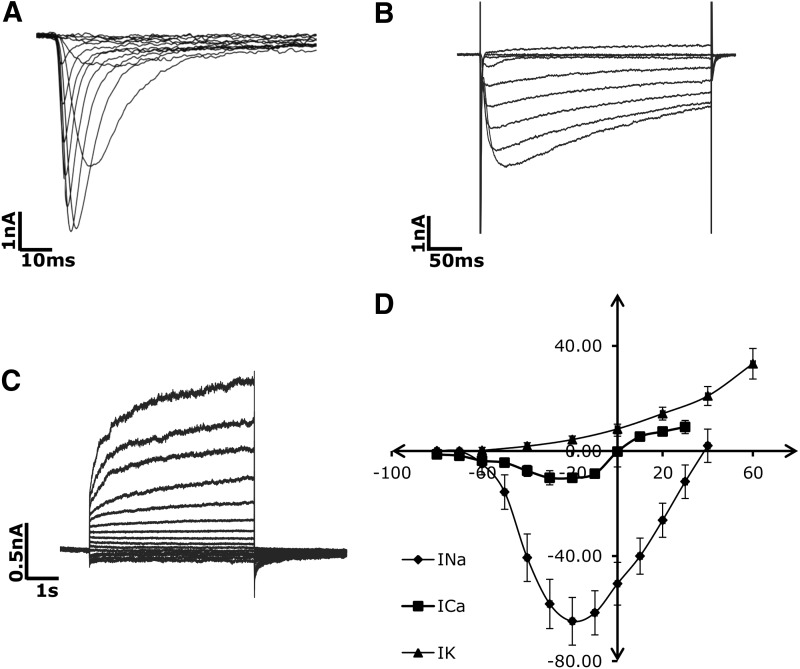
Automated electrophysiological analysis of HUES7 hESC-CMs in the voltage clamp mode. Representative raw data traces of **(A)** I_Na_, **(B)** I_Ca_, and **(C)** I_K_ currents in HUES7-CMs. The maximal current densities for the three currents were determined to be **−**64.8 ± 9.1 pA/pF (at **−**20 mV, *n* = 31), **−**10.2 ± 3.8 pA/pF (at **−**30 mV, *n* = 12), and 20.9 ± 3.6 pA/pF (at +40 mV, *n* = 24) respectively. **(D)** Averaged I/V (current-voltage) diagrams of the I_Na_, I_Ca_, and I_K_ currents in HUES7-CMs.

The ability of the Patchliner to implement stepped depolarizing pulses on parallel channels gives it the potential to carry out rapid measurement of ion channel densities. We initially used HUES7-CMs to define the necessary protocols to unveil I_Na_, I_Ca,L_, and I_K_ currents. To record I_Na_, 20 ms long depolarizing pulses between −70 and +40 mV were applied in 10 mV increments from a holding potential of −80 mV. I_Na_ was first observed at −60 mV, progressively increased to reach a maximal level at −20 mV, and progressively decreased on further depolarization (Fig. 5A). To record I_Ca_, cells were first held at a potential of −80 mV, after which a 3s long prepulse of −50 mV was applied to voltage inactivate Na^+^ and any T-type Ca^+2^ channels that may be present. Then, 200 ms long depolarizing pulses between −70 and +30 mV were applied in 10 mV increments. I_Ca_ was first observed at −60 mV, progressively increased to reach a maximal level at −30 mV, and progressively decreased on further depolarization (Fig. 5B). To record I_K_, 200 ms long depolarizing pulses between −60 and +60 mV were applied in 20 mV increments, from a holding potential of −80 mV. I_K_ was first observed at −40 mV, and progressively increased to reach a maximal level at +60 mV (Fig. 5C). Maximal peak I_Na_, I_Ca_, and I_K_ normalized to cell capacitance (*n* = 67, mean = 24.38 ± 2.32 pF) were 64.83 ± 9.11 pA/pF (*n* = 31, −20 mV), 10.2 ± 2.73 pA/pF (*n* = 12, −30 mV), and 20.95 ± 3.59 pA/pF (*n* = 24, +40 mV) respectively. V_1/2_ values for the same were calculated to be −34.76 ± 9.52 mV, −33.4 ± 10.2 mV, and −20.6 ± 12.5 mV respectively.

**FIG. 6.**
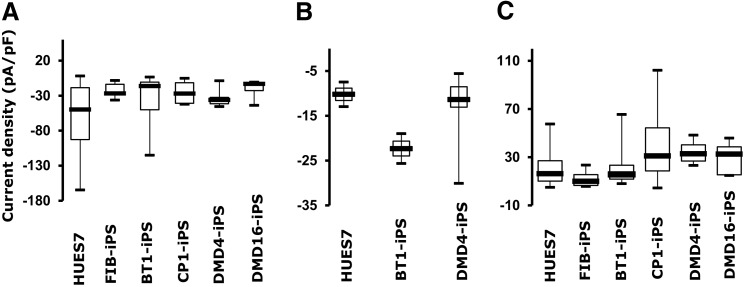
A comparison of the electrophysiological properties of hPSC-CMs using the Patchliner. Box plots demonstrating the distribution of **(A)** I_Na_, **(B)** I_Ca_, and **(C)** I_K_ current maxima, at **−**20,**−**30, and +40 mV respectively, across six hPSC lines. These cell lines included HUES7-fibroblast-derived “healthy” FIB-hiPSCs, dental pulp-derived BT1-hiPSCs, Duchenne muscular dystrophy (DMD)-afflicted DMD4- and DMD6-hiPSCs, catecholaminergic polymorphic ventricular tachycardia (CPVT)-afflicted CP1-hiPSCs, and “healthy” HUES7-hESCs. The maximal current densities were shown to range from **−**19.6 ± 4.5 pA/pF to **−**37.3 ± 8.9 pA/pF, **−**12.8 ± 2.4 pA/pF to **−**22.3 ± 3.3 pA/pF, and 12.2 ± 4.1 pA/pF to 40.3 ± 6.4 pA/pF for I_Na_, I_Ca_, and I_K_ respectively. Error bars represent standard error of mean (*n* = 5 for each cell line).

Using the parameters defined for HUES7-CMs, current–voltage curves and current densities were determined for five additional hPSC lines. These included hiPSC lines produced by reprogramming embryonic fibroblasts or patient-derived dental pulp cells from healthy individuals, or from skin fibroblasts derived from patients carrying mutations causing CPVT and DMD. The maximal current densities for cardiomyocytes derived from these cell lines ranged from −19.6 ± 4.5 pA/pF to −37.3 ± 8.9 pA/pF, −12.8 ± 2.4 pA/pF to −22.3 ± 3.3 pA/pF, and 12.2 ± 4.1 pA/pF to 40.3 ± 6.4 pA/pF for I_Na_, I_Ca,_ and I_K_, respectively (Table 6, *n* = 5 for each cell line, Fig. 6).

**Table 6.** Current Densities of Human Pluripotent Stem Cell-Cardiomyocytes as Determined by Automated Planar Patch Clamp

	*Current density (pA/pF)*					
*Current (test voltage)*	*HUES7-hESC*	*FIB-iPS*	*BT1-iPS*	*CP1-iPS*	*DMD4-iPS*	*DMD16-iPS*
Na (−20 mV)	−64.8 ± 9.1	−32.5 ± 16.1	−37.3 ± 8.9	−25.4 ± 9.4	−32.9 ± 6.4	−19.6 ± 4.5
Ca (−30 mV)	−10.2 ± 3.8	n.d.	−22.3 ± 3.3	n.d.	−12.8 ± 2.4	n.d.
K (40 mV)	20.9 ± 3.6	12.2 ± 4.1	22.5 ± 6.1	40.3 ± 6.4	34.3 ± 5.6	29.4 ± 6.2

DMD, Duchenne muscular dystrophy; hESCs, human embryonic stem cells; n.d., not determined.

Single-factor ANOVA tests were conducted across the different lines, for each channel current. In case of the I_Na_ current, there were significant differences in mean current densities across the six lines at *P* < 0.05 [*F*(5,65) = 2.73, *P* = 0.027]. Post hoc comparisons using the Tukey HSD test indicated that the maximal I_Na_ current density of HUES7-CMs was significantly different than those of cardiomyocytes derived from FIB-iPS [absolute difference (ABS) = 42.33], BT1-iPS (ABS = 27.53), CP1-iPS (ABS = 39.4), DMD4-iPS (ABS = 31.94), and DMD16-iPS (ABS = 45.21). In case of the I_Ca_ maximal current densities, there were no significant differences across the HUES7-, BT1-iPS, and DMD4-iPS-derived cardiomyocytes at *P* < 0.05 [*F*(2,10) = 1.92, *P* = 0.197]. Similarly, no significant differences were found in maximal I_K_ current densities across the six lines at *P* > 0.05 [*F*(5,54) = 2.32, *P* = 0.056]. Taken together, these results suggest that the stem cell-derived cardiomyocytes were a heterogeneous group of cells with variable cardiac phenotypes, as has been reported by several other research groups [39].

### Efficient pharmacological screening of hPSC-CMs by automated planar patch clamp

Next, the effects of different ion channel modulators on hPSC-CM currents were determined using the Patchliner. In brief, peak I_Na_, I_Ca_, and I_K_ currents were measured at pulses of −20, −30, and +40 mV respectively. Steady state recordings were conducted for 3 min before the application of escalating doses of their respective blockers. Peak current amplitude was averaged from four voltage pulses for each drug concentration applied and for the baseline recordings. Dose–response data were fitted to the Hill Equations and IC_50_ values determined for each channel blocker tested.

**FIG. 7.**
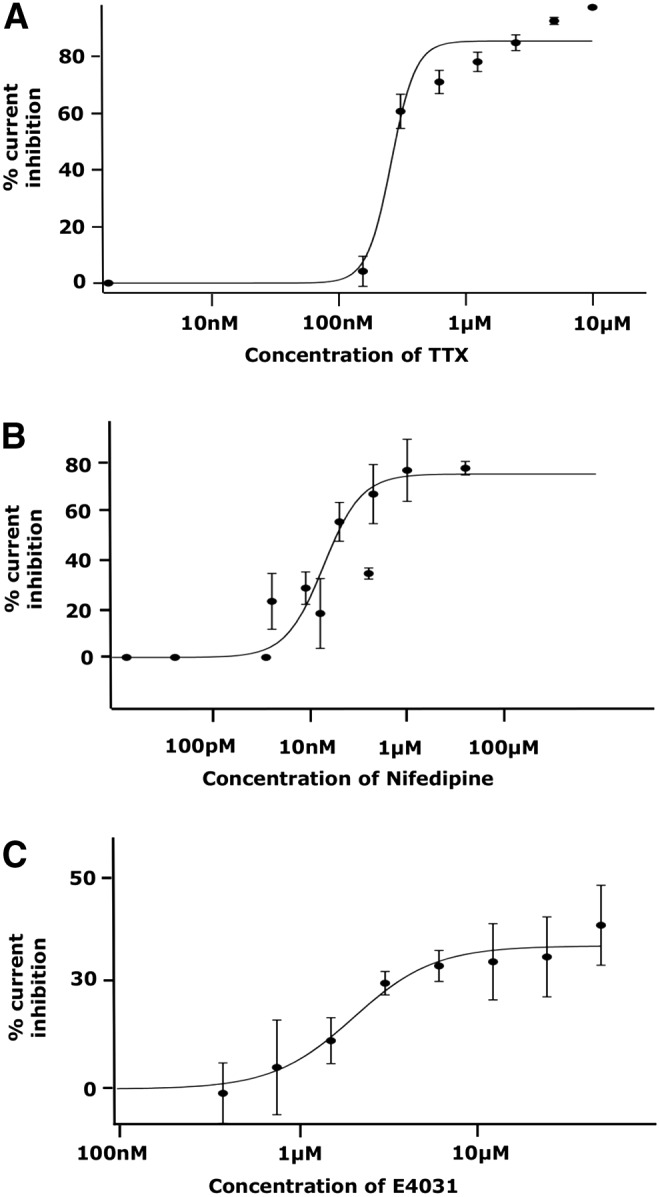
Efficient pharmacological screening of hPSC-CMs by automated planar patch clamp. Patch clamp analysis showing dose-dependent inhibition of the **(A)** I_Na_, **(B)** I_Ca_, and **(C)** I_K_ currents, in hPSC-CMs, on treatment with increasing concentrations of TTX, nifedipine, and terfenadine respectively. IC_50_ values of 289.8 ± 31.9 nM, 1.4 ± 1.5 μM, and 2 ± 1.4 μM were calculated for TTX, nifedipine, and E-4031 respectively. Error bars represent standard deviation of the mean (*n* = 5 for each drug).

TTX, a potent blocker of the I_Na_ channels, caused a concentration-dependent inhibition of the I_Na_ current of the hPSC-CMs, with an IC_50_ of 289.8 ± 31.9 nM (Fig. 7A, *n* = 5). Increasing concentrations of nifedipine, an L-type Ca^+2^ channel blocker, inhibited the current amplitude of the I_Ca.L_ channels in a dose-dependent manner, with an IC_50_ of 1.4 ± 1.5 μM (Fig. 7B, *n* = 5). Finally, E-4031—a selective I_Kr_ (rapidly activating delayed rectifier potassium channel) blocker—was applied to the cells in increasing doses to check for a reduction in total I_K_. A concentration-dependent inhibition of the I_K_ current was observed and an IC_50_ of 2 ± 1.4 μM was calculated (Fig. 7C, *n* = 5). Thus, this feasibility study showed the suitability of the Patchliner APC system for the electrophysiological and pharmacological analyses of hPSC-CMs.

## Discussion

In this study, we demonstrated the potential of the Patchliner planar patch clamp platform for hPSC-CM-based medium-throughput drug safety and efficacy testing. To achieve this, we developed a cell preparation protocol that allowed the dissociation of EB and monolayer cultures of cardiomyocytes into single cells, while maintaining their viability and membrane integrity. When analyzed on the Patchliner, the freshly dissociated cardiomyocytes demonstrated AP generation, depolarizing Na^+^ and Ca^+2^ currents, and repolarizing K^+^ currents, proving their suitability for use in disease modeling and high-throughput drug screening.

In conventional patch clamping the cell is accessed by means of a glass pipette that is manually brought into contact with its membrane [38], while in planar patch clamp the traditional glass pipette is replaced by a hole at the bottom of a well through which an applied negative pressure patches the cell membrane [30]. This allows automation of the patch clamp process that reduces the time and labor costs associated with conventional patch clamp, and parallelization that drastically improves screening efficiency and data output [29].

Today there exist several APC platforms, including the IonWork Quattro (MDS), PatchXpress (Molecular Devices Corporation), Patchliner (Nanion), SynchroPatch (Nanion), QPatch (Sophion), and Flyscreen (Flyion), differing in their technical specifications and level of automation [29]. Initially developed for the analysis of recombinant cell lines used for heterologous channel expression, these are only now starting to be trialled for use with more “complex” cells like stem cell-derived cardiomyocytes [31,37,40] and neurons [37,41].

Cell preparation is a key factor in the performance of an APC device, as it has a direct impact on catch rates, seal qualities and observed current characteristics. Specifically, these systems require high-density, homogenous, single-cell suspensions for optimal performance [29]. Conventional APC systems, like the QPatch [40] and the PatchExpress [31], use between 8,000 and 150,000 PSC-CMs per recording site at a density of 0.5–5 million cells/mL. Such high cell numbers are required to maximize the probability of positioning a cell over the recording aperture, and in many cases increases in catch rates have been shown to positively correlate with increases in cell densities [42].

With recent improvements in cardiac differentiation protocols, hPSC-CMs can be produced in large numbers, but with high reagent/labor costs. To illustrate, the loading of 48 recording wells of an APC, at a density of 150,000 cells/well, would require 7.2 million cells at a cost of USD$7,200 when purchasing them from a commercial source. To reduce costs we used a specially developed “cell stacking” protocol that reduces cell usage to 300–2,000 cells/recording site [37], making it more economical for use in the high-throughput screening of hPSC-CMs.

To obtain single-cell suspensions, cardiomyocytes cultures are usually dissociated with enzymes like trypsin, as gentler nonenzymatic harvest protocols produce cell clusters that block recording sites, lowering the productivity of the system. However, trypsin has been previously shown to induce proteome alterations of mammalian cell membranes, with a subset of these proteins remaining dysregulated even after a 24 h recovery in fresh culture media [36]. Trypsin has also been shown to affect the integrity of membrane phospholipids [43], which could lead to reduced catch rates, low seal resistances, and poor channel activity. In conventional patch clamp, cells are typically seeded onto coverslips following dissociation, and kept in culture for at least 72 h before use [38], which gives them time to recover from enzyme treatment. However, in case of APC, cells are analyzed, in suspension, within a few hours of dissociation and so the dissociation protocol used strongly impacts seal rates and observed current outputs.

In this study, we overcame these problems by developing a two-step cell dissociation protocol. First, intact EB/monolayer cultures of hPSC-CMs were dissociated into single cells using a trypsin-based dissociation method, and then reseeded at low densities onto gelatine-coated tissue-culture plastic. Next, following a week of recovery in culture, the attached single cells were reharvested using a gentle and rapid accutase-assisted cold dissociation protocol for immediate analysis on the Patchliner. This two-step protocol yielded catch rates of 81.25% ± 6.68% and seal resistances of up to 2 GΩ, which are a considerable improvement over previous studies using APC devices like the PatchXpress that reported seal resistances of up to only 200 MΩ using iCell hiPSC-CMs [31].

The cardiomyocytes used in our experiments were derived by both the monolayer and EB-routes. Regular manual patch clamp analysis was used to first validate the suitability of the cardiomyocytes for further use. These experiments revealed atrial-, nodal-, and ventricular-like APs based on generally accepted classification criteria [15,21]. Compared with adult human cardiomyocytes, our cells have hyperpolarized RMPs, low upstroke velocities, smaller APAs, and shorter APs. This is indicative of a fetal/neonatal phenotype and appears to be characteristic of hPSC-CMs [14,16,31,34].

When making automated recordings from our hPSC-CMs in current clamp mode, we found spontaneously beating cells to show erratic beat patterns that were unsuitable for analysis. This could be due to the cells being in suspension and at 4°C immediately before analysis. Though this instability could be controlled by the use of stimulation protocols, we found that different hPSC-CMs displayed different stimulation thresholds. This meant each well required the individual definition of stimulatory conditions, which detracted from the automated nature of the platform. Thus, we would suggest that data collection from hPSC-CMs on the Patchliner platform is most effective in voltage clamp rather than current clamp mode. We also found the cells to express higher APAs and lower RMPs using APC, which could be due to differences in ionic conditions, temperatures, and holding potentials between the two platforms.

On automated voltage clamp analysis, our hPSC-CMs displayed current densities and activation kinetics similar to that of other hPSC-CMs and fetal/neonatal cardiomyocytes. They also predictably responded to the three ion channel blockers used in the study. However, our voltage clamp experiments did not include the recording of cellular APs beforehand to allow for data classification into atrial-, ventricular-, and nodal-like subtypes. This could explain the wide variations in current densities within the same line. Further studies also need to be performed to identify whether the observed inter-line variations in current densities is due to differences in the expression of ion channels, or due to the presence of the channels in different conductance states.

It is important to note that when comparing our data with the existing literature, we found big differences in ionic conditions, experimental protocols, tissue heterogeneity, and disease status across studies [31,44–49], which raises the question whether results are reproducible across research groups [50]. Nevertheless, currently available high-efficiency cardiac differentiation protocols [12,34] in concert with the high-content screening abilities of APCs provide a platform for reliable and efficient drug candidate safety profiling and efficacy testing in industrial and academic settings.

In conclusion, this study compared the electrophysiological properties of cardiomyocytes derived from six different hPSC lines on an APC system. We presented an optimized cell preparation protocol that markedly improved the productivity of the Patchliner when using hPSC-CMs, thus making APC recordings compatible with the study of expensive and heterogeneous cell preparations. This will substantially enhance the feasibility of using APC platforms for drug development using hPSC-CMs.

## Supplementary Material

Supplemental data

Supplemental data

Supplemental data

Supplemental data

Supplemental data

Supplemental data

Supplemental data

Supplemental data

Supplemental data
