# Supplementary material for: Automated Electrophysiological and Pharmacological Evaluation of Human Pluripotent Stem Cell-Derived Cardiomyocytes
Source: Stem Cells Dev. 2016 Feb 23;25(6):439–52. doi: 10.1089/scd.2015.0253 (PMC4790208; doi:10.1089/scd.2015.0253)
Supplement: Supplemental data [file Supp_Fig1.pdf]

Supplementary Data

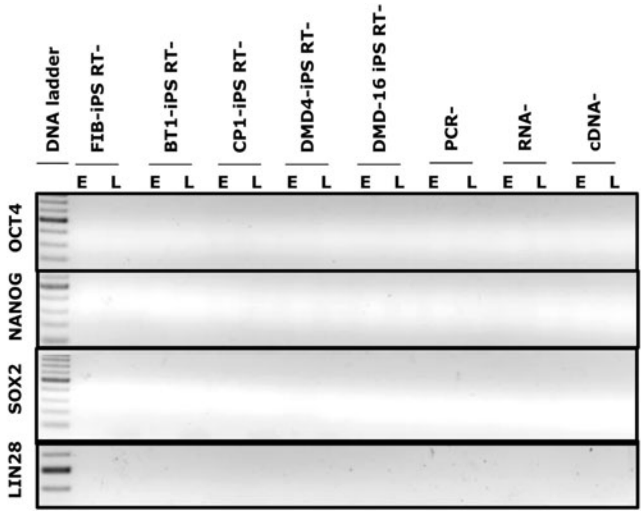

SUPPLEMENTARY FIG. S1. Demonstration of the silencing of lentiviral transgenes and the reactivation of endogenous pluripotency genes in the five hiPSC lines used in the study (negative controls). Gel images of negative controls used in the RT-PCR experiments for demonstration of the silencing of lentiviral ''L'' transgenes and the activation of endogenous ''E'' pluripotency genes in the FIB-iPS, BT1-iPS, CP1-iPS, DMD4-iPS, and DMD16-iPS lines. These include RT-, PCR-, RNA-, and cDNA- controls. DMD, Duchenne muscular dystrophy; FIB, HUES7-fibroblast-derived; hiPSC, human induced pluripotent stem cells.
