# Supplementary material for: Automated Electrophysiological and Pharmacological Evaluation of Human Pluripotent Stem Cell-Derived Cardiomyocytes
Source: Stem Cells Dev. 2016 Feb 23;25(6):439–52. doi: 10.1089/scd.2015.0253 (PMC4790208; doi:10.1089/scd.2015.0253)
Supplement: Supplemental data [file Supp_Fig2.pdf]

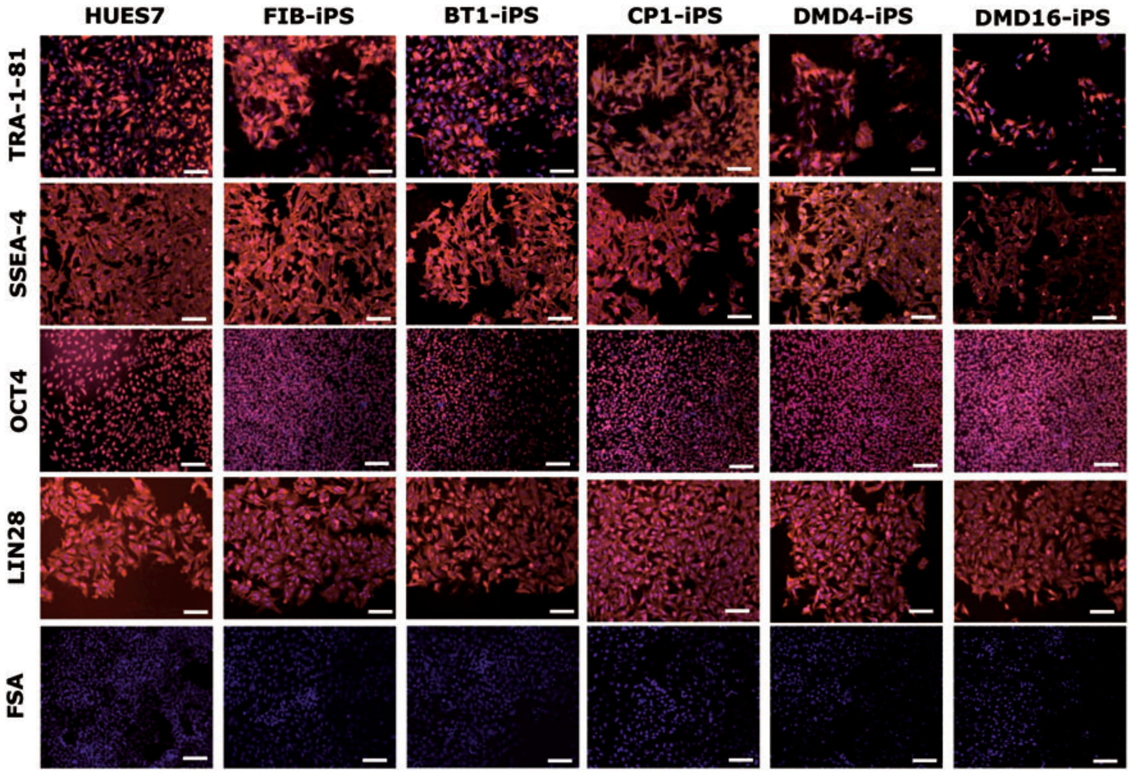

SUPPLEMENTARY FIG. S2. Detection of pluripotency marker expression in the hiPSC lines by immunofluorescence Immunofluorescence analysis of HUES7 hESCs and FIB-, BT1-, CP1-, DMD4- and DMD16-hiPSCs, showing them to stain negative for the fibroblast-specific marker FSA, and stain positive for the pluripotency markers TRA-1-81, SSEA-4, OCT-4, and LIN28. Scale bars represent 65 μm. hESCs, human embryonic stem cells.
