# Supplementary material for: Automated Electrophysiological and Pharmacological Evaluation of Human Pluripotent Stem Cell-Derived Cardiomyocytes
Source: Stem Cells Dev. 2016 Feb 23;25(6):439–52. doi: 10.1089/scd.2015.0253 (PMC4790208; doi:10.1089/scd.2015.0253)
Supplement: Supplemental data [file Supp_Fig3.pdf]

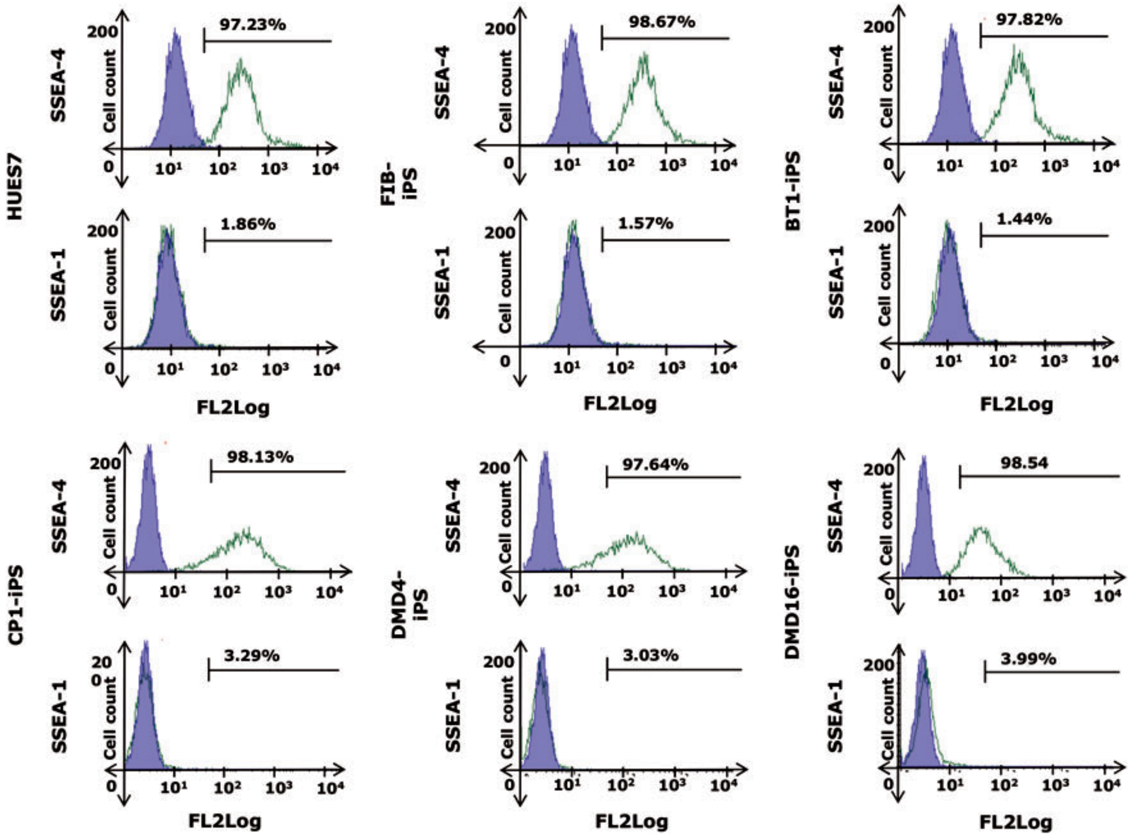

SUPPLEMENTARY FIG. S3. Detection of pluripotency marker expression by flow cytometry. Flow cytometry analysis of HUES7 hESCs and FIB-, BT1-, CP1-, DMD4-, and DMD16-hiPSCs, showing them to be positive for the pluripotency marker SSEA-4 and negative for the differentiation marker SSEA-1.
