# Supplementary material for: Automated Electrophysiological and Pharmacological Evaluation of Human Pluripotent Stem Cell-Derived Cardiomyocytes
Source: Stem Cells Dev. 2016 Feb 23;25(6):439–52. doi: 10.1089/scd.2015.0253 (PMC4790208; doi:10.1089/scd.2015.0253)
Supplement: Supplemental data [file Supp_Video1.zip › Supp_Video1.pdf]

SUPPLEMENTARY VIDEO S1. A representative video of beating sheets of hPSC-CMs derived using the monolayer method of cardiac differentiation. hPSC-CMs, human pluripotent stem cell-derived cardiomyocytes.
