# Supplementary material for: Automated Electrophysiological and Pharmacological Evaluation of Human Pluripotent Stem Cell-Derived Cardiomyocytes
Source: Stem Cells Dev. 2016 Feb 23;25(6):439–52. doi: 10.1089/scd.2015.0253 (PMC4790208; doi:10.1089/scd.2015.0253)
Supplement: Supplemental data [file Supp_Video2.zip › Supp_Video2.pdf]

SUPPLEMENTARY VIDEO S2. A representative video of a beating cluster of hPSC-CMs derived using the embryoid body method of cardiac differentiation. hPSC-CMs, human pluripotent stem cell-derived cardiomyocytes.
