# Supplementary material for: Automated Electrophysiological and Pharmacological Evaluation of Human Pluripotent Stem Cell-Derived Cardiomyocytes
Source: Stem Cells Dev. 2016 Feb 23;25(6):439–52. doi: 10.1089/scd.2015.0253 (PMC4790208; doi:10.1089/scd.2015.0253)
Supplement: Supplemental data [file Supp_Fig5.pdf]

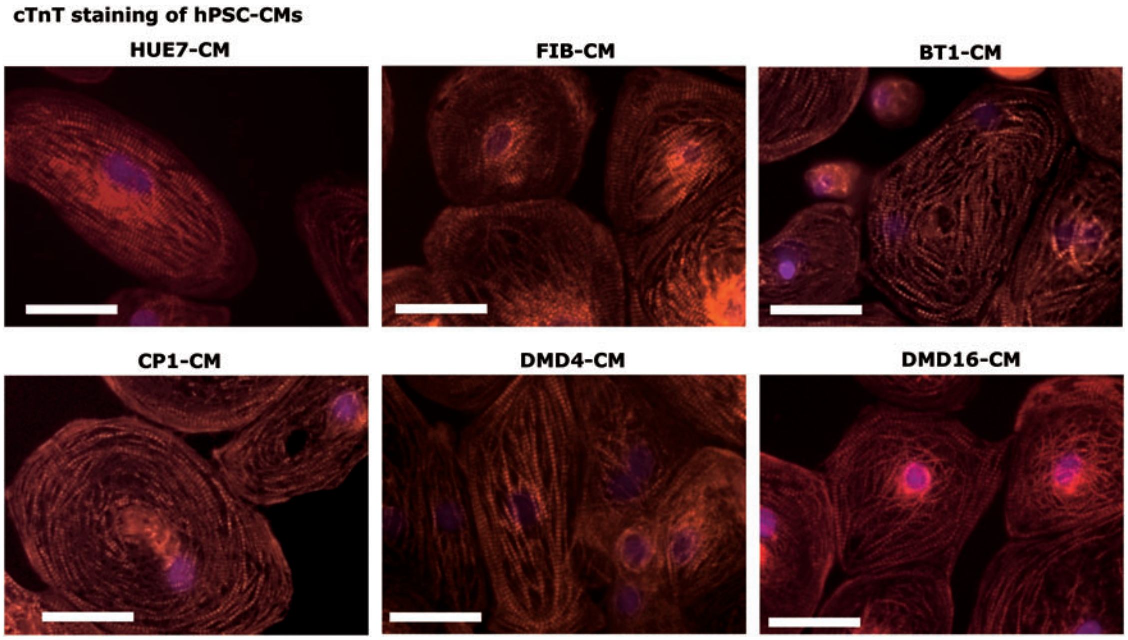

SUPPLEMENTARY FIG. S5. Detection of cTnT expression in hPSC-CMs by immunofluorescence. Immunofluorescence images of cardiomyocytes derived from HUES7 hESCs and FIB-, BT1-, CP1-, DMD4-, and DMD16-hiPSCs, showing positive cardiac troponin T staining highlighting characteristic cardiac muscle striations. Scale bars represent 32 μm. cTnT, cardiac troponin T.
