# Supplementary material for: Automated Electrophysiological and Pharmacological Evaluation of Human Pluripotent Stem Cell-Derived Cardiomyocytes
Source: Stem Cells Dev. 2016 Feb 23;25(6):439–52. doi: 10.1089/scd.2015.0253 (PMC4790208; doi:10.1089/scd.2015.0253)
Supplement: Supplemental data [file Supp_Fig6.pdf]

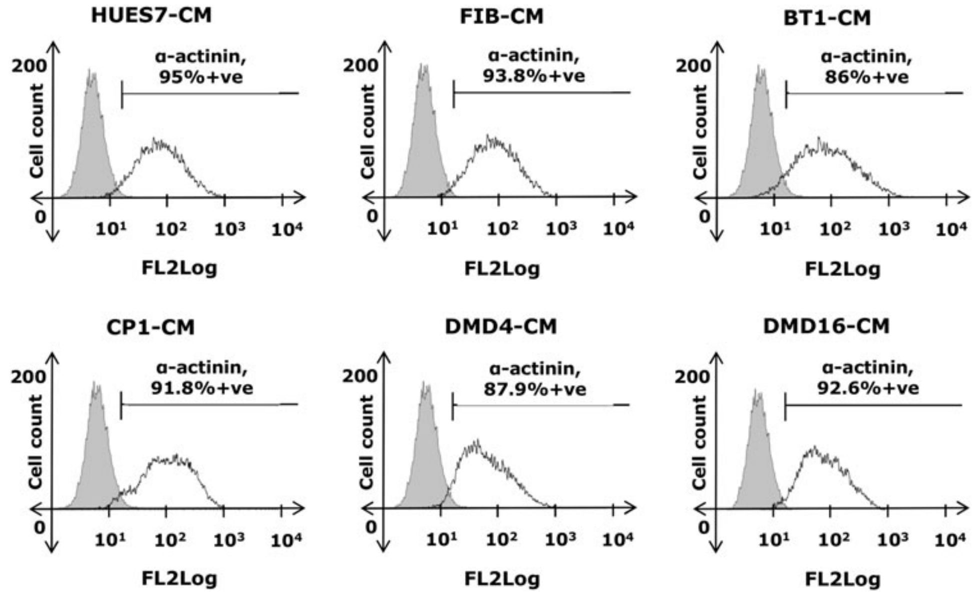

SUPPLEMENTARY FIG. S6. Assessment of purity of hPSC-CM preparations by flow cytometry. Flow cytometry data of cardiomyocytes derived from HUES7 hESCs and FIB-, BT1-, CP1-, DMD4-, and DMD16-hiPSCs demonstrating cardiac purities of 91.2% ± 1.4%.
