# Supplementary material for: Automated Electrophysiological and Pharmacological Evaluation of Human Pluripotent Stem Cell-Derived Cardiomyocytes
Source: Stem Cells Dev. 2016 Feb 23;25(6):439–52. doi: 10.1089/scd.2015.0253 (PMC4790208; doi:10.1089/scd.2015.0253)
Supplement: Supplemental data [file Supp_Figs2-6.pdf]

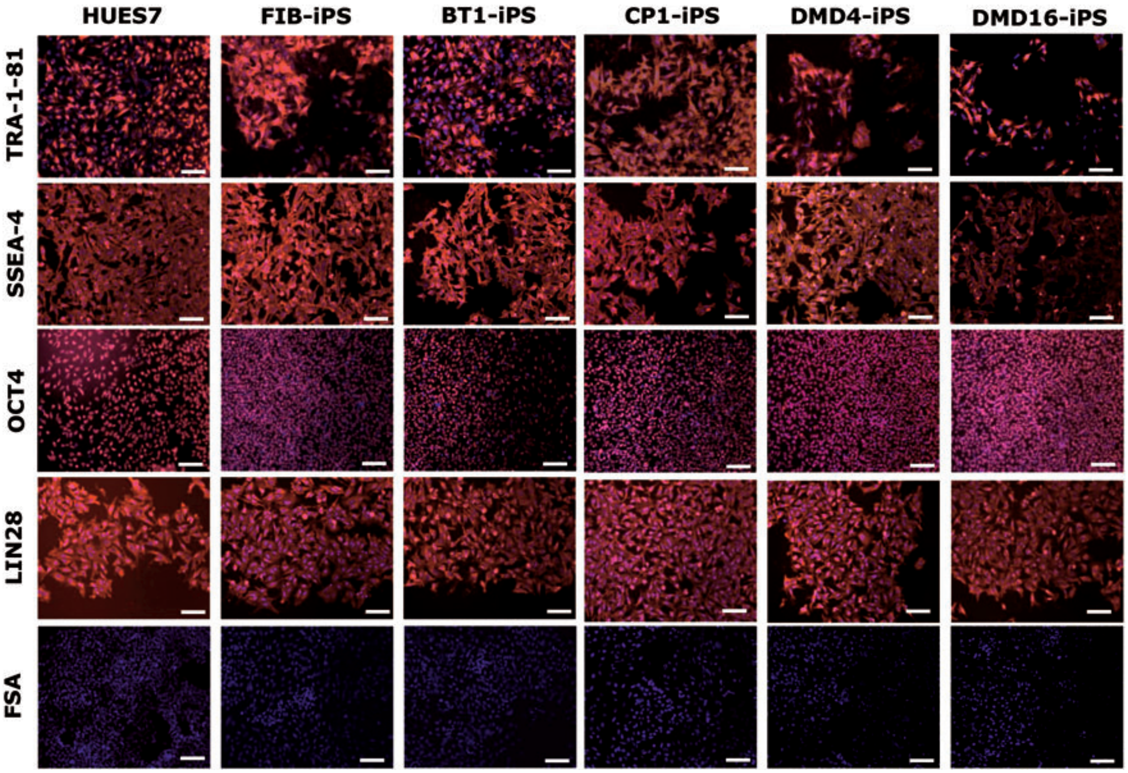

SUPPLEMENTARY FIG. S2. Detection of pluripotency marker expression in the hiPSC lines by immunofluorescence Immunofluorescence analysis of HUES7 hESCs and FIB-, BT1-, CP1-, DMD4- and DMD16-hiPSCs, showing them to stain negative for the fibroblast-specific marker FSA, and stain positive for the pluripotency markers TRA-1-81, SSEA-4, OCT-4, and LIN28. Scale bars represent 65 μm. hESCs, human embryonic stem cells.

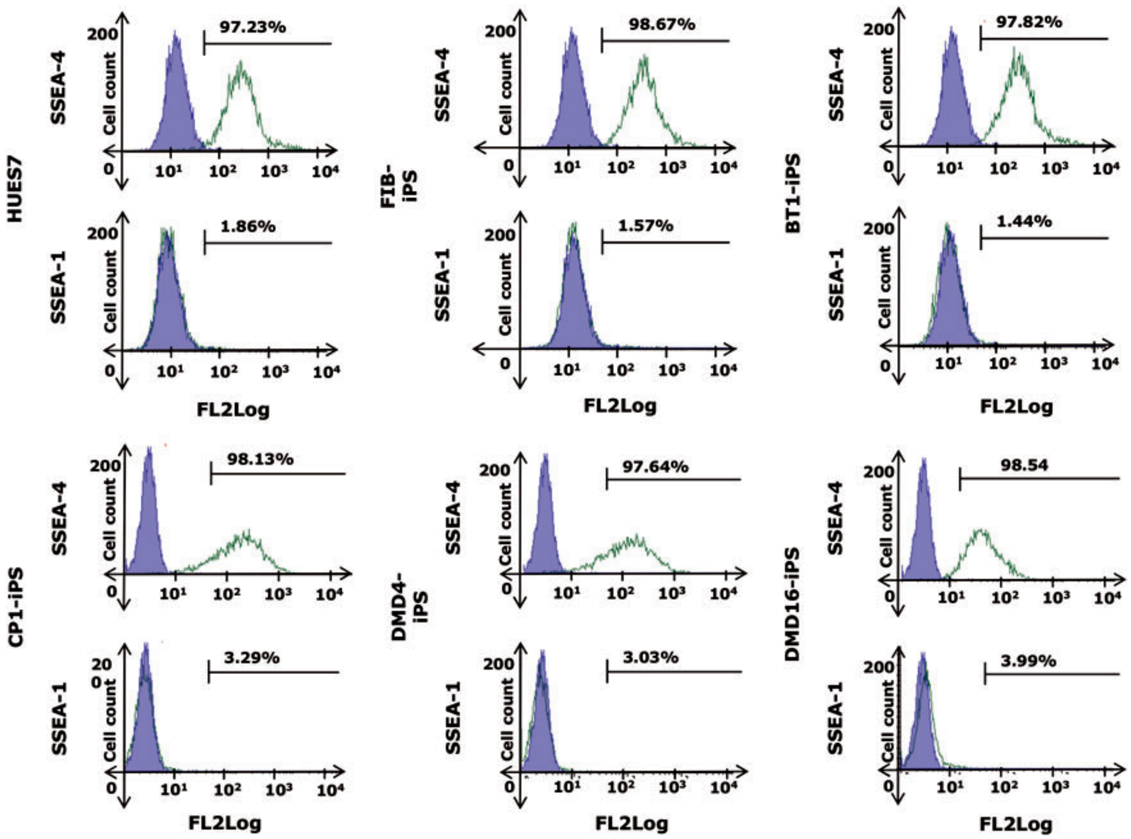

SUPPLEMENTARY FIG. S3. Detection of pluripotency marker expression by flow cytometry. Flow cytometry analysis of HUES7 hESCs and FIB-, BT1-, CP1-, DMD4-, and DMD16-hiPSCs, showing them to be positive for the pluripotency marker SSEA-4 and negative for the differentiation marker SSEA-1.

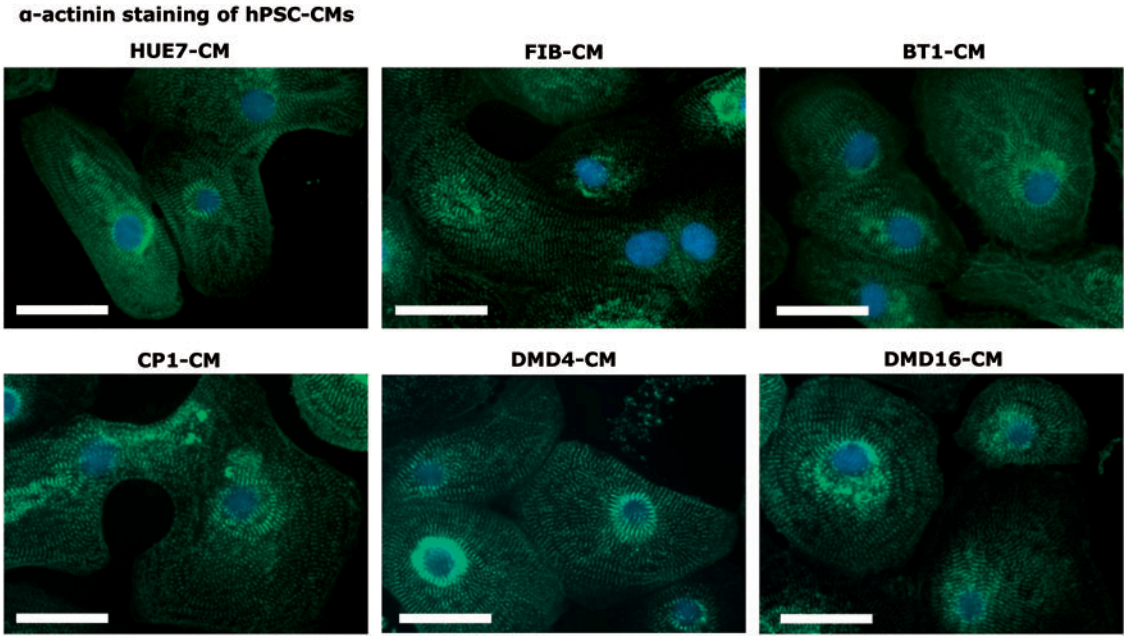

SUPPLEMENTARY FIG. S4. Detection of α-actinin expression in hPSC-CMs by immunofluorescence. Immunofluorescence images of cardiomyocytes derived from HUES7 hESCs and FIB-, BT1-, CP1-, DMD4-, and DMD16-hiPSCs, showing positive cardiac α-actinin staining highlighting characteristic cardiac muscle striations. Scale bars represent 32 μm. hPSC-CMs, human pluripotent stem cell-derived cardiomyocytes.

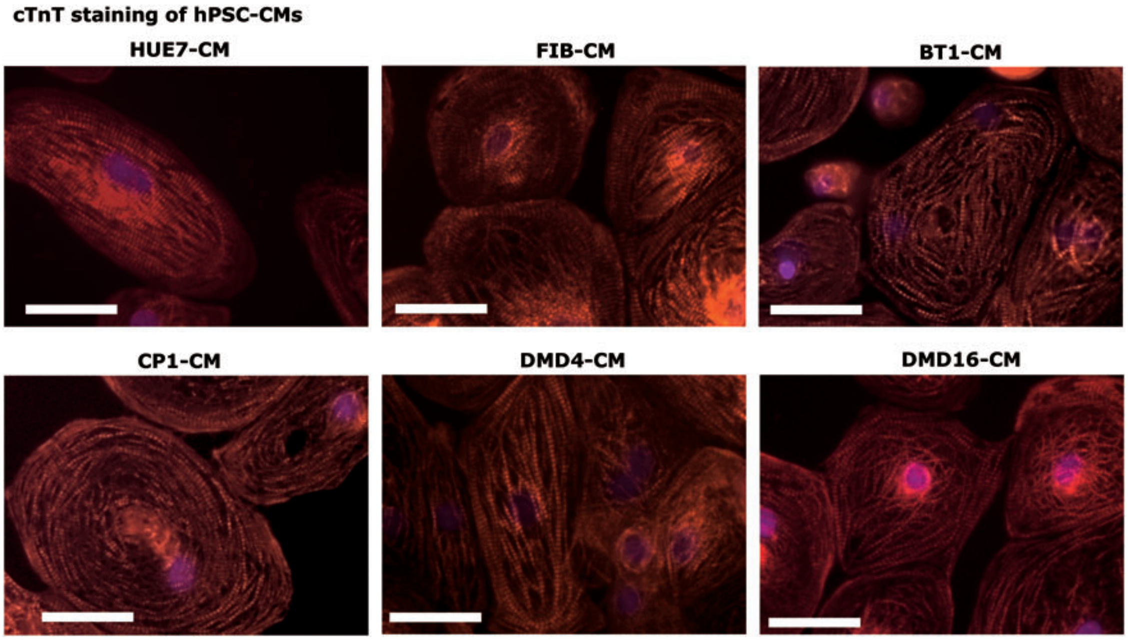

SUPPLEMENTARY FIG. S5. Detection of cTnT expression in hPSC-CMs by immunofluorescence. Immunofluorescence images of cardiomyocytes derived from HUES7 hESCs and FIB-, BT1-, CP1-, DMD4-, and DMD16-hiPSCs, showing positive cardiac troponin T staining highlighting characteristic cardiac muscle striations. Scale bars represent 32 μm. cTnT, cardiac troponin T.

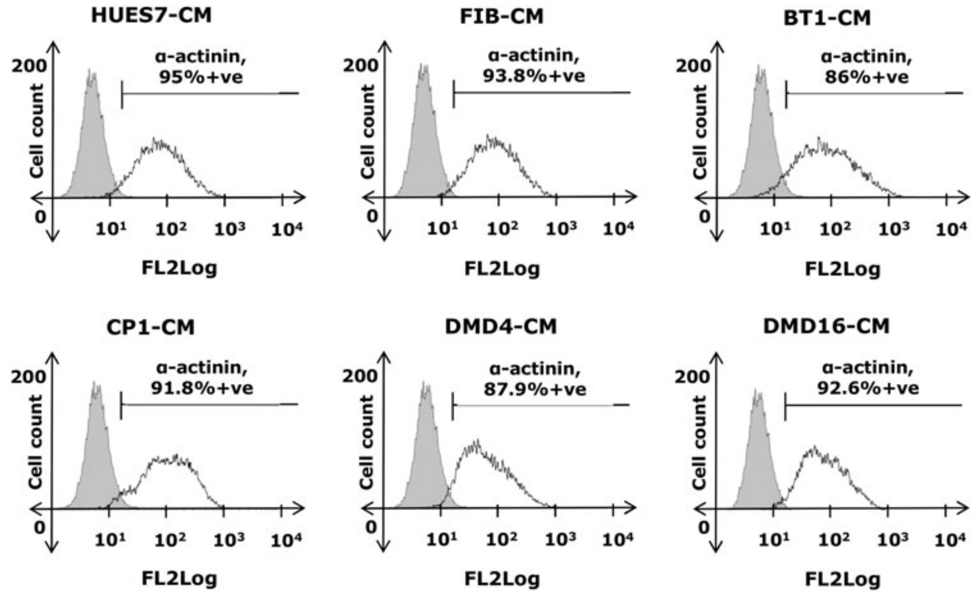

SUPPLEMENTARY FIG. S6. Assessment of purity of hPSC-CM preparations by flow cytometry. Flow cytometry data of cardiomyocytes derived from HUES7 hESCs and FIB-, BT1-, CP1-, DMD4-, and DMD16-hiPSCs demonstrating cardiac purities of 91.2% ± 1.4%.
